# Overnight stiffness index from finger photoplethysmography in relation to markers of cardiovascular risk and vascular ageing

**DOI:** 10.1007/s00380-025-02537-3

**Published:** 2025-03-14

**Authors:** Henrik Hellqvist, Hermine Rietz, Ludger Grote, Jan Hedner, Dirk Sommermeyer, Thomas Kahan, Jonas Spaak

**Affiliations:** 1 Division of Cardiovascular Medicine, Department of Clinical Sciences, Danderyd Hospital, Karolinska Institutet, Stockholm, Sweden; 2 Department of Internal Medicine and Clinical Nutrition, Center for Sleep and Vigilance Disorders, Sahlgrenska Academy, University of Gothenburg, Gothenburg, Sweden

**Keywords:** Ambulatory blood pressure monitoring, Hypertension, Photoplethysmography, Pulse wave analysis, Arterial stiffness, Cardiovascular risk

## Abstract

Wearable technology, such as photoplethysmography (PPG), enables easily accessible individual health data with the potential for improved risk assessment. We hypothesized that the overnight stiffness index (OSI), derived from nocturnal finger PPG, could be used to assess cardiovascular risk and vascular ageing. Subjects with confirmed or suspected hypertension (*n* = 79, 56 males) underwent simultaneous ambulatory blood pressure monitoring (ABPM) and overnight sleep polygraphy with a continuous PPG registration. Overnight PPG-based pulse propagation time was used to calculate OSI. Associations between OSI and markers of cardiovascular risk, blood pressure, and indices of arterial stiffness, as indicators of vascular ageing, were assessed. Subjects were stratified into low and high OSI (according to median, 10.9 m/s). SCORE2/SCORE2-OP and Framingham risk scores were calculated. The high OSI group had higher SCORE2/SCORE2-OP (9.5 [5.5;12.5] vs 5.0 [4.0;6.5]), and OSI correlated with SCORE2/SCORE2-OP and Framingham risk score (*r*_s_ = 0.40 and *r*_s_ = 0.41; both *P* < 0.01). Indices of arterial stiffness were increased in the high OSI group including ABPM awake and asleep pulse pressures (59 ± 14 vs 50 ± 9 mmHg, *P* < 0.01, and 54 ± 14 vs 45 ± 7 mmHg, *P* < 0.001), and ambulatory arterial stiffness index (0.47 ± 0.12 vs 0.37 ± 0.11, *P* < 0.001), respectively. OSI correlated with 24-h and asleep pulse pressure also after adjusting for confounders. OSI was related to systolic ABPM (awake *r* = 0.42, asleep *r* = 0.55; both *P* < 0.001) and diastolic ABPM (asleep *r* = 0.36, *P* < 0.01). OSI, a novel PPG-based measure of nocturnal arterial stiffness, correlates with established cardiovascular risk scores and with blood pressure-derived indices of vascular ageing. This simple method may facilitate cardiovascular risk assessment using readily available medical and wearable consumer devices.

**Supplementary Information:**

The online version contains supplementary material available at 10.1007/s00380-025-02537-3.

## Introduction

Assessment of cardiovascular disease risk is essential for identification of individuals at risk and to implement proper preventive measures [1–3]. Risk prediction models have been developed to guide clinical decisions in the apparently healthy general population. These include the SCORE2, SCORE2-OP, and the Framingham risk score [4–6], which are based on basic clinical characteristics such as age, gender, systolic blood pressure, cholesterol levels, and smoking status.

Measurable characteristics that can improve risk assessment beyond traditional risk scores include the presence of impaired renal function, increased C-reactive protein, coronary artery calcium content, and carotid or femoral artery plaques. Another measurable risk characteristic is arterial stiffness [1, 7], which is a salient feature in early vascular ageing, and a major contributor to the development of hypertension and cardiovascular disease [8]. Measures of aortic and large artery stiffness are independent markers for future cardiovascular events and mortality [9, 10]. Increased pulse pressure, a consequence of increased arterial stiffness, obtained from office blood pressure or ambulatory blood pressure monitoring (ABPM), is associated with adverse outcomes [11]. Also, ambulatory arterial stiffness index (AASI), which is obtained by analyzing the linear relationships between individual systolic and diastolic blood pressure measurements from ABPM, is considered a marker of aortic and large artery stiffness and can predict cardiovascular morbidity and mortality [12].

Evidently, current cardiovascular risk assessment relies on the use of laboratory tests, blood pressure measurements and specific imaging and vascular assessment methods. Given the importance of cardiovascular prevention, there is an unmet need for novel and accessible methods to facilitate and improve cardiovascular risk assessment. Photoplethysmography (PPG) is an optical method that measures instant blood volume changes in the skin and the underlying tissue and vasculature, which can be used for pulse wave analysis [13–15]. PPG is commonly used to measure oxygen saturation levels in the arterial blood and pulse rate, but is becoming progressively used in consumer devices such as smart watches, smart rings, and fitness trackers, and has lately been studied for evaluation of arterial stiffness and vascular ageing [16].

One feature of the PPG waveform that reflects aortic stiffness is the pulse propagation, or peak-to-peak, time (PPT). PPT is the time delay between the first and the second peaks of the PPG curve, which is considered to represent the transit time of the pressure wave from the heart to the peripheral sites of reflection and back again [17, 18]. This distance is proportional to body height, and a height-adjusted index of large artery stiffness can be calculated as height/PPT, the stiffness index (SI) [18–20]. SI is related to aortic pulse wave velocity (PWV) [19–21] and cardiovascular risk [22, 23]. SI improves risk stratification and is an independent predictor of cardiovascular outcomes and mortality [24].

**Fig. 1:**
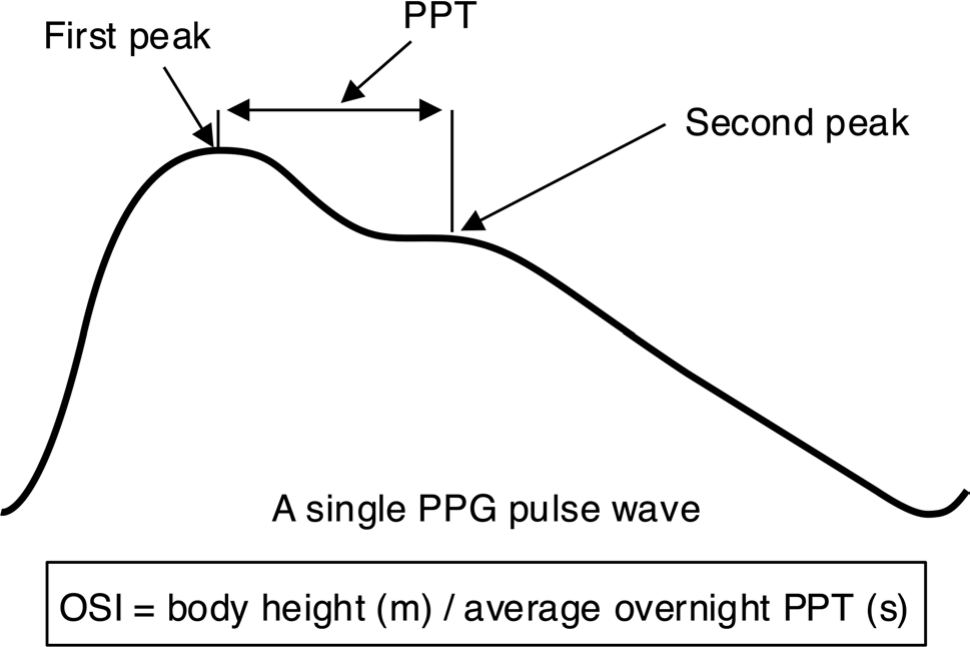
Pulse propagation time (PPT) is the time delay between the first and the second peaks of the photoplethysmography (PPG) pulse wave. Overnight stiffness index (OSI) is a height-adjusted measure of large artery stiffness derived from average overnight PPT

Most previous studies on PPT and SI have utilized short daytime PPG recordings [16]. However, it is plausible that PPG recordings in the resting state during the night are more robust, less influenced by disturbing factors, and provide a more reliable assessment [25]. Thus, we have previously shown that overnight PPT (OPPT) in patients with suspected obstructive sleep apnoea (OSA) contributed to the stratification of cardiovascular risk, when being part of risk models based on overnight PPG [26, 27]. Furthermore, OPPT was related to office blood pressure and a diagnosis of hypertension, and OPPT was longest in the deepest state of sleep, suggesting a possible added value of assessments overnight [28]. However, until now the height-adjusted PPG-based overnight SI (OSI) has not been studied. Furthermore, previous work included only people undergoing sleep studies for suspected OSA. Thus, for the current study we hypothesized that OSI may provide an improved PPG-based measure to assess cardiovascular risk and vascular ageing in a general population of hypertensive subjects without known OSA (Fig. 1).

## Materials and methods

### Subjects

This study is based on subjects referred to the Department of Cardiology, Danderyd University Hospital, Stockholm (Sweden) between 2009 and 2016 for a 24-h ABPM due to suspected or confirmed hypertension. Eligible subjects without known OSA underwent simultaneous ABPM and overnight sleep polygraphy. From this larger cohort, 79 subjects had complete overnight registrations of both PPG and ABPM and were included in this study. The study was conducted in accordance with the Declaration of Helsinki and was approved by the Regional Ethics Review Board (2008/1675–31), Stockholm (Sweden). Written informed consent was obtained from each subject prior to study participation.

### Assessment of clinical characteristics

All subjects provided information on medical history and smoking habits. Further information on concomitant conditions, diseases, and medications was obtained from electronic medical records. At the study visit, height and weight were measured. Office blood pressure was measured by guideline-recommended standard procedures in the seated position with the auscultatory method.

Routine biochemistry was determined by standard methods. Lipid status was obtained after overnight fasting. Low-density lipoprotein cholesterol (LDL) levels were determined by Friedewald's formula. Estimated glomerular filtration rate (eGFR) was calculated by the 2021 CKD-EPI equation. Mild, moderate, and severe OSA were defined as an apnoea–hypopnea index (AHI) of ≥ 5 to < 15, ≥ 15 to < 30, and ≥ 30 events/h, respectively.

### Stiffness indices from ambulatory blood pressure recordings

ABPM was performed using Spacelabs ABP 90217A monitors (Spacelabs Healthcare, Snoqualmie, Washington, USA) during 24 h with recordings every 20 min on the non-dominant arm. The subject-reported bedtime and wake-up times were used to define the periods of being awake and asleep. Rules to define valid recordings were based on the modified version of the Casadei procedure with no further editing [29]. Pulse pressure values were calculated as the mean of systolic minus diastolic blood pressure, for each period (24-h, awake, and asleep), and blood pressure dipping as (mean day blood pressure—mean night blood pressure)/mean day blood pressure × 100) for systolic and diastolic blood pressure. AASI was calculated as 1—the linear regression slope of each recorded diastolic on systolic blood pressure during the full 24-h recording [30].

### Stiffness index from overnight photoplethysmography

A sleep diagnostics device (SOMNOcheck micro CARDIO, Löwenstein Medical Technology GmbH, Hamburg, Germany) with built-in finger photoplethysmography and continuous pulse wave analysis (ChipOx: Corscience GmbH & Co. KG, Erlangen, Germany) at 100 Hz was performed simultaneously with the ABPM (on the contralateral arm). Shorter periods with artefacts were automatically discarded by the device. All subjects had > 3 h valid PPG recordings. OPPT was calculated by the device, defined as the time (in ms) from the first peak to the second peak in the pulse waveform, calculated beat by beat, and averaged for the whole sleep recording. OSI was defined as body height (in m) divided by OPPT (in s). The subjects were grouped into low and high OSI (below and above median, 10.9 m/s). This value is comparable to the threshold of > 10 m/s for carotid–femoral pulse wave velocity (in people 50–60 years of age) considered to define hypertension-mediated organ damage [1].

### Cardiovascular risk scores

SCORE2 and SCORE2-OP for moderate risk region and algorithms for ages 40–69 and 70–89 years, respectively, and the Framingham risk score were calculated [4–6] (Supplementary Information about cardiovascular risk scores). Missing information on lipid status, age, systolic blood pressure outside algorithm recommendations, and the presence of diabetes mellitus resulted in SCORE2/SCORE2-OP values for 60 subjects and Framingham risk score values for 64 subjects.

### Statistical analysis

Descriptive data are presented as mean values ± standard deviation (SD), median values with interquartile ranges, and proportions, as appropriate. Comparisons between groups for continuous variables were assessed by Welch’s *t* test, Wilcoxon’s rank sum test, or Kruskal–Wallis test, as appropriate, and for categorical variables by the Chi-squared test or, when frequencies were below 5, by Fisher’s exact test. Between-group differences were corrected for multiple comparisons using the Bonferroni method. Associations were evaluated by Pearson’s or Spearman’s rank correlation analyses, and, by multiple linear regression models, as appropriate. For Pearson’s correlation analysis, assumptions were assessed through diagnostic plots, and correlations were checked to persist overall, after removal of occasional outliers, and correlation differences were tested using Williams’ test for significant correlations. The multiple linear regression models included the potential confounders: age, sex, body mass index, diabetes, and smoking history, due to their established associations with cardiovascular risk and arterial stiffness. Model assumptions were checked through inspection of diagnostic plots and calculation of variance inflation factors. The models were evaluated after adjustment for multiple testing by the Hommel method, and in sensitivity analyses after removal of potential outliers and influential observations. A two-sided probability level (*P*) < 0.05 was taken as significant. Analyses were performed using the software R, version 4.4.1 (R Foundation for Statistical Computing, Vienna, Austria).

## Results

### Study subjects

The clinical characteristics of the study participants are presented in Table 1. Subjects in the high OSI group were approximately nine years older and had higher cardiovascular risk according to SCORE2/SCORE2-OP.

**Table 1:** Clinical characteristics of the study population

	All	Low OSI (< 10.9 m/s)	High OSI (≥ 10.9 m/s)	*P*	*Q*
n	79	39	40	–	–
Age, years	58.1 ± 10.8	53.4 ± 10.9	62.6 ± 8.7	< 0.001	0.002
Sex, male	56 (71%)	26 (67%)	30 (75%)	0.57	> 0.99
Height, cm	175.3 ± 9.4	176.4 ± 10.0	174.1 ± 8.8	0.28	> 0.99
Body mass index, kg/m^2^	28.1 ± 4.0	28.7 ± 4.4	27.5 ± 3.6	0.20	> 0.99
Smoking history	28 (36%)	13 (34%)	15 (38%)	0.95	> 0.99
Diabetes mellitus	7 (9%)	4 (10%)	3 (8%)	0.71	> 0.99
Antihypertensive medication	40 (51%)	17 (44%)	23 (58%)	0.31	> 0.99
Statin treatment	26 (33%)	11 (28%)	15 (38%)	0.52	> 0.99
Total cholesterol, mmol/l	5.30 ± 1.09	5.33 ± 1.21	5.28 ± 0.97	0.85	> 0.99
LDL cholesterol, mmol/l	3.34 ± 1.00	3.39 ± 1.04	3.29 ± 0.96	0.68	> 0.99
HDL cholesterol, mmol/l	1.32 ± 0.36	1.26 ± 0.39	1.38 ± 0.32	0.19	> 0.99
eGFR, ml/min/1.73m^2^	89 ± 17	91 ± 17	86 ± 17	0.25	> 0.99
SCORE2/SCORE2-OP, %	6.5 [4.5;10.8]	5.0 [4.0;6.5]	9.5 [5.5;12.5]	< 0.001	0.005
Framingham risk score, %	17 [10;27]	14 [7;22]	21 [13;31]	0.011	0.17
Office SBP, mmHg	147 ± 19	143 ± 17	151 ± 21	0.10	> 0.99
Office DBP, mmHg	90 ± 11	92 ± 9	88 ± 11	0.10	> 0.99

Values presented as mean values ± SD, median values [interquartile range], or n (%), with *P* level of significance between groups and *Q*, showing adjusted *P* values after correction for multiple comparisons

*OSI* overnight stiffness index, *eGFR* estimated glomerular filtration rate, *SBP* systolic blood pressure, *DBP* diastolic blood pressure. Diabetes mellitus includes both type 1 and 2

### Overnight stiffness index and cardiovascular risk scores

**Fig. 2:**
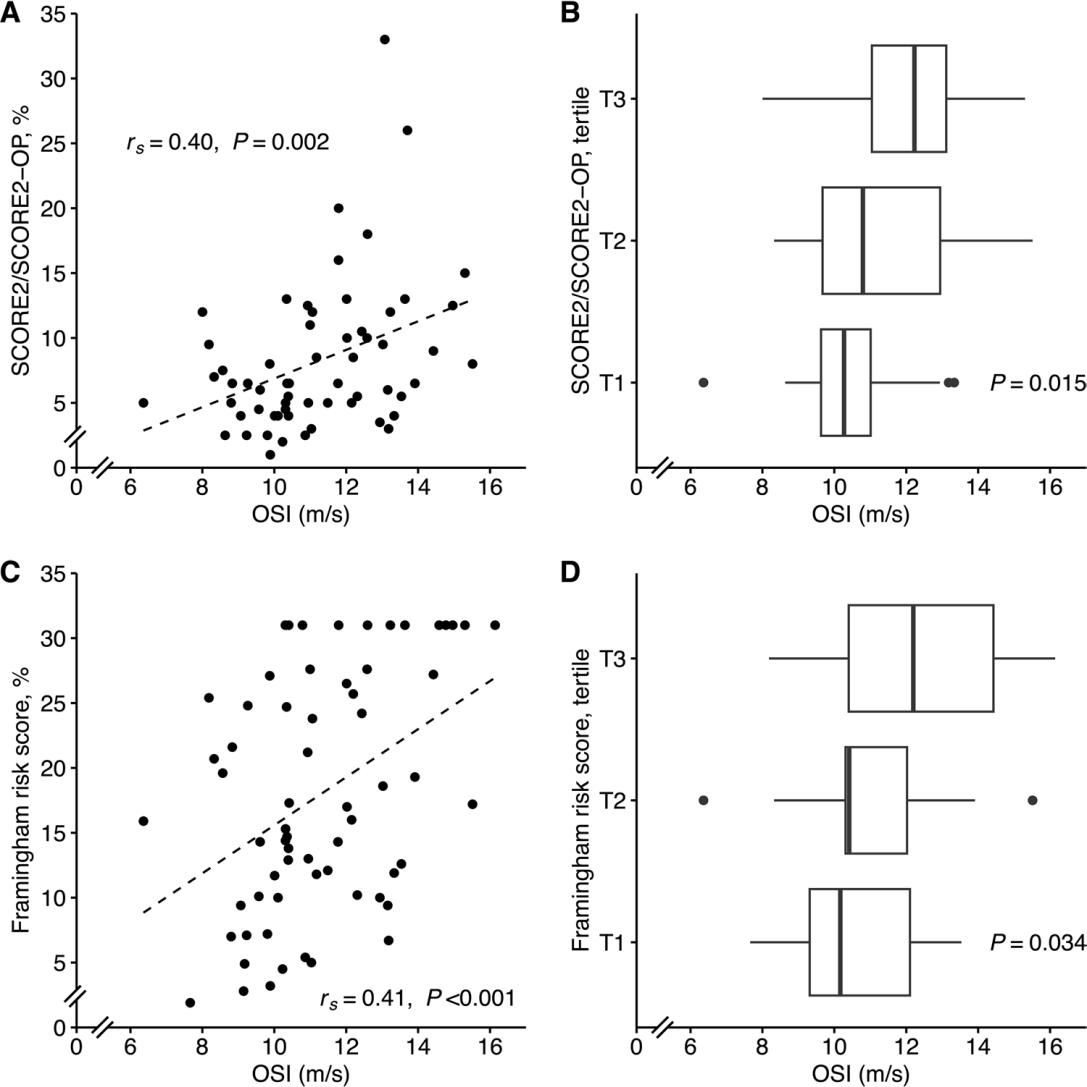
Overnight stiffness index (OSI) in relation to cardiovascular risk scores. **A** and **C** Spearman’s rank correlation analysis with *r*_s_ coefficient and *P* value for each scatter plot. The dashed line represents a simple linear regression line. **B** and **D** *P* values from Kruskal–Wallis test for comparison between tertiles, and box plots where vertical lines indicate the median, the lower and upper edges correspond to the first and third quartiles, whiskers extend to the highest/lowest values which are within ± 1.5 × interquartile range, and data outside the whiskers are considered as outliers and plotted as dots. Subjects with Framingham risk score > 30% were approximated to 31%. T, tertile. **A**, **B**: *n* = 60. **C**, **D**: *n* = 64

Higher OSI was associated with an increased cardiovascular risk, as shown by the difference of OSI between the risk score tertiles and by the associations with these scores (Fig. 2). OSI was also associated with age alone, as a marker of cardiovascular risk (*r* = 0.45, *P* < 0.001).

### Overnight stiffness index and indices of arterial stiffness

**Fig. 3:**
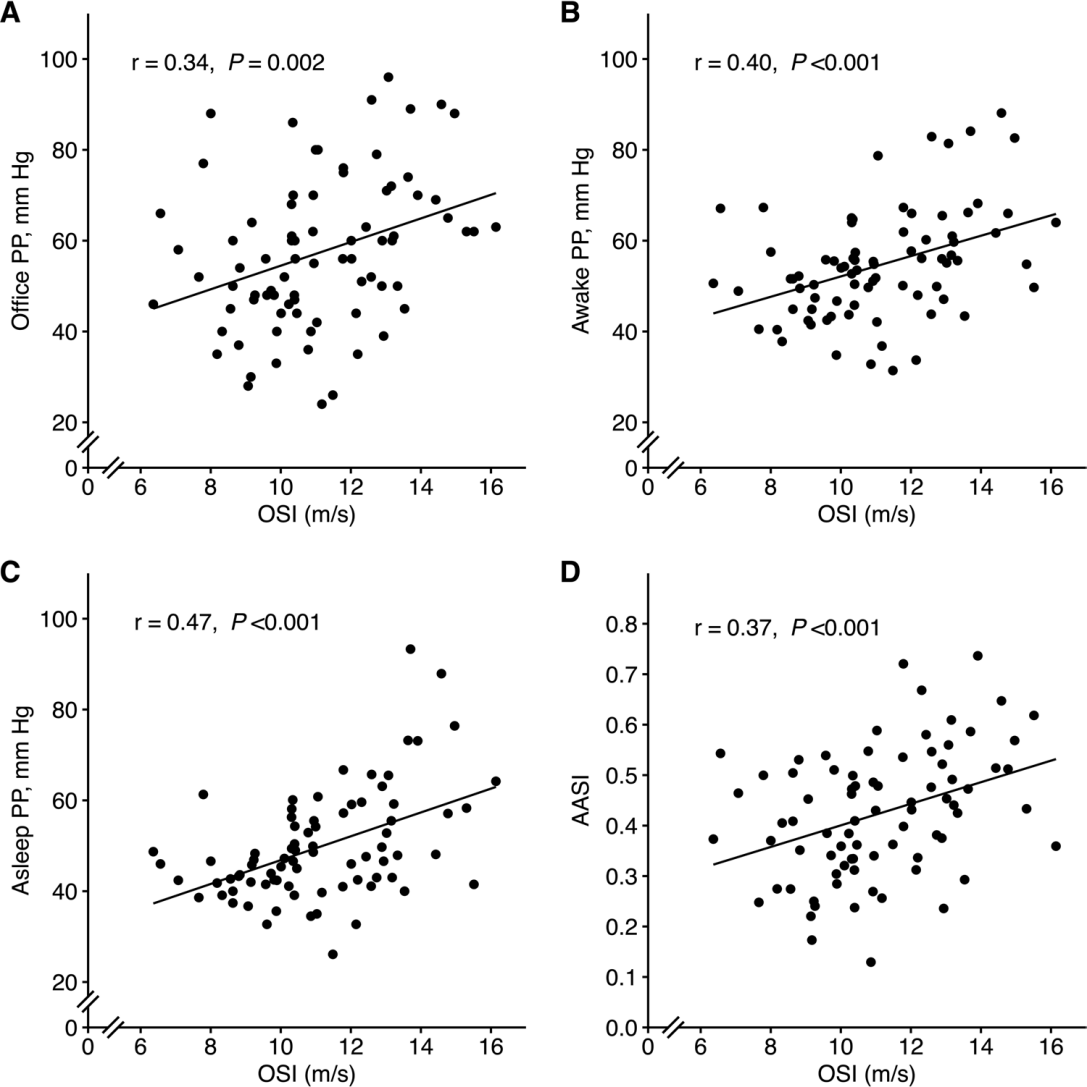
Overnight stiffness index (OSI) in relation to blood pressure-derived indices of vascular ageing and arterial stiffness. **A** Office PP, **B** awake PP, **C** sleep PP, **D** AASI. *PP* pulse pressure, *AASI* ambulatory arterial stiffness index

Several blood pressure-derived indices of arterial stiffness (i.e. office pulse pressure and pulse pressures by ABPM, and AASI) were higher in the high OSI group, as compared to the low OSI group (Table 2), and were all related to OSI (Fig. 3; and for 24-h pulse pressure: *r* = 0.44, *P* < 0.001). After controlling for potential confounders, OSI remained independently associated with 24-h and asleep pulse pressure (Supplementary Table S1). In a sensitivity analysis removing outliers and influential observations, these associations persisted, and also the associations between OSI and office pulse pressure and awake pulse pressure became significant (data not shown).

**Table 2:** Ambulatory blood pressure measurements and indices of vascular ageing and arterial stiffness

	All	Low OSI (< 10.9 m/s)	High OSI (≥ 10.9 m/s)	*P*	*Q*
*n*	79	39	40	–	–
24-h SBP, mmHg	135 ± 15	129 ± 9	140 ± 17	< 0.001	0.007
Awake SBP, mmHg	141 ± 15	136 ± 10	146 ± 17	0.002	0.027
Asleep SBP, mmHg	121 ± 16	114 ± 10	128 ± 18	< 0.001	0.001
24-h DBP, mmHg	82 ± 8	80 ± 7	83 ± 9	0.17	> 0.99
Awake DBP, mmHg	86 ± 9	85 ± 7	87 ± 10	0.35	> 0.99
Asleep DBP, mmHg	72 ± 9	69 ± 8	74 ± 9	0.019	0.25
Systolic dipping, %	14 ± 7	16 ± 6	12 ± 7	0.026	0.34
Diastolic dipping, %	17 ± 8	19 ± 7	15 ± 8	0.029	0.38
Office PP, mmHg	57 ± 17	52 ± 14	63 ± 17	0.002	0.030
24-h PP, mmHg	53 ± 12	49 ± 8	57 ± 13	< 0.001	0.012
Awake PP, mmHg	55 ± 12	50 ± 9	59 ± 14	0.002	0.024
Asleep PP, mmHg	50 ± 12	45 ± 7	54 ± 14	< 0.001	0.008
AASI	0.42 ± 0.13	0.37 ± 0.11	0.47 ± 0.12	< 0.001	0.005

Values presented as mean values ± SD, (%), with *P* level of significance between groups and *Q*, showing adjusted *P* values after correction for multiple comparisons

*OSI* overnight stiffness index, *SBP* systolic blood pressure, *DBP* diastolic blood pressure, *PP* pulse pressure, *AASI* ambulatory arterial stiffness index

### Overnight stiffness index and ambulatory blood pressure levels

**Fig. 4:**
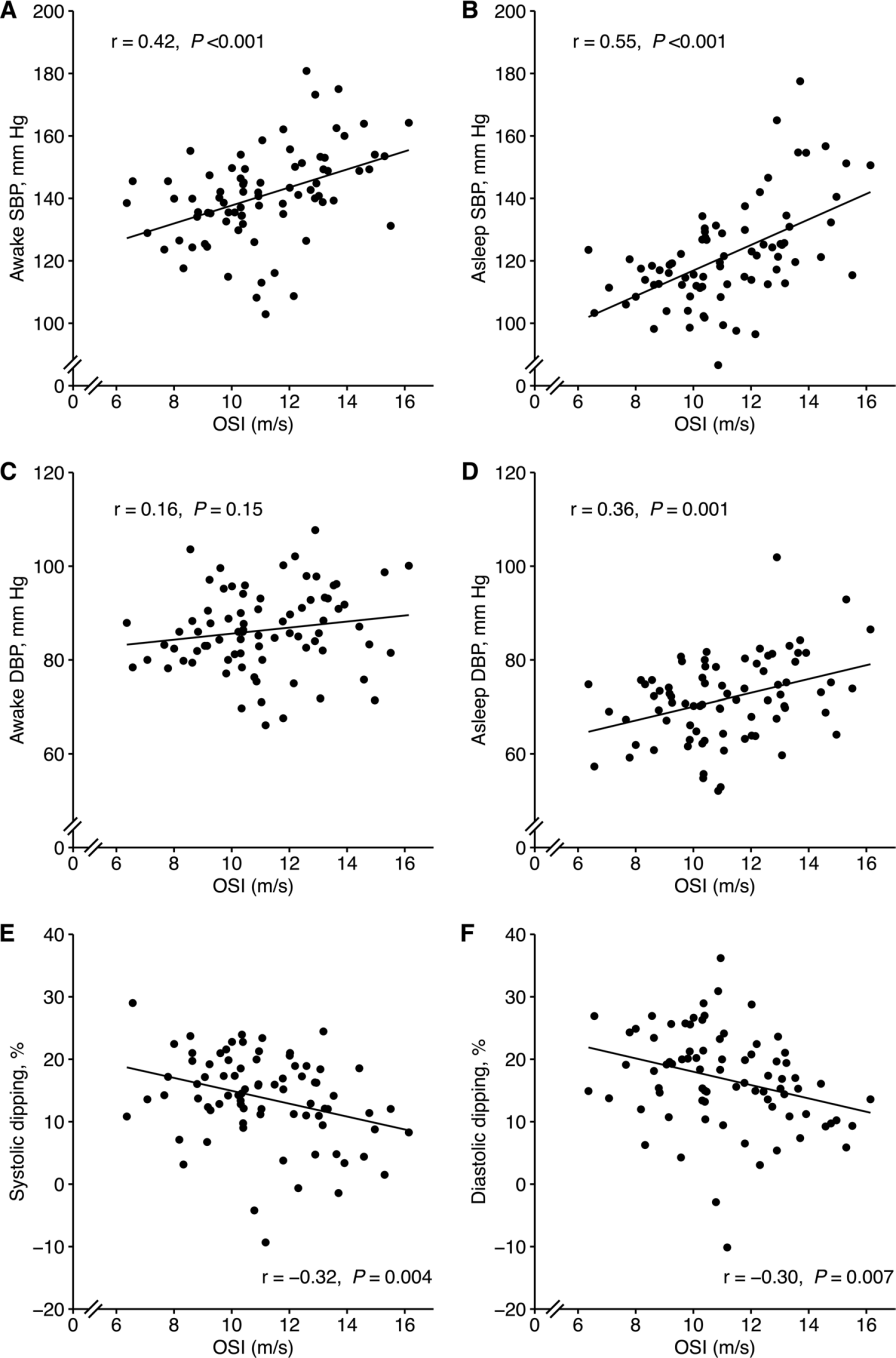
Overnight stiffness index (OSI) in relation to blood pressure variables. **A** Awake and **B** asleep SBP, **C** awake and **D** asleep DBP, **E** systolic and **F** diastolic dipping. Pearson correlation analysis with r coefficient and *P* value for each scatter plot. *SBP* systolic blood pressure, *DBP* diastolic blood pressure

The high OSI group generally had higher systolic ABPM values than the low OSI group (Table 2). OSI was associated with blood pressure levels, with stronger associations for systolic than for diastolic blood pressure, and for the sleep period, as compared to the awake period (Fig. 4). Blood pressure dipping was inversely associated with OSI (Fig. 4). OSI remained independently associated with 24-h, awake, and asleep systolic ABPM levels, after controlling for potential confounders (Supplementary Table S2), and remained significant in a sensitivity analysis removing outliers and influential observations (data not shown).

### Overnight stiffness index and obstructive sleep apnoea

Among the 71 subjects with available data on AHI, 20 (28%) had no OSA, 27 (38%) mild, 21 (30%) moderate, and 3 (4%) severe OSA. OSI, but not OPPT, pulse pressures, or AASI, differed in subjects with OSA (Supplementary Table S3). OSI was associated with the number of respiratory events during sleep (AHI, *r*_s_ = 0.40, *P* < 0.001); however, after controlling for potential confounders, OSI did not retain a significant association with AHI (Supplementary Table S4).

### Overnight stiffness index versus overnight pulse propagation time

Compared to OPTT, OSI showed stronger numeric correlations with ambulatory blood pressure levels, blood pressure dipping, blood pressure-derived indices of arterial stiffness, and markers of cardiovascular risk (Table 3). OPPT correlated with participant height (*r* = 0.33, *P* = 0.003), as expected.

**Table 3:** OSI and OPPT in relation to ABPM metrics, indices of arterial stiffness, and risk scores

	OSI		OPPT		Difference		
	Correlation coefficient	*P*	Correlation coefficient	*P*	Absolute	Relative	*P*
24-h SBP, mmHg	0.48	< 0.001	0.39	< 0.001	+ 0.09	+ 24%	0.012
Awake SBP, mmHg	0.42	< 0.001	0.34	0.002	+ 0.08	+ 23%	0.046
Asleep SBP, mmHg	0.55	< 0.001	0.43	< 0.001	+ 0.12	+ 27%	0.001
24-h DBP, mmHg	0.23	0.041	0.14	0.21	–	–	–
Awake DBP, mmHg	0.16	0.15	0.09	0.43	–	–	–
Asleep DBP, mmHg	0.36	0.001	0.24	0.033	+ 0.12	+ 50%	0.002
Systolic dipping, %	0.32	0.004	0.23	0.039	+ 0.09	+ 37%	0.035
Diastolic dipping, %	0.30	0.007	0.22	0.049	+ 0.08	+ 35%	0.057
Office PP, mmHg	0.34	0.002	0.28	0.011	+ 0.06	+ 20%	0.16
24-h PP, mmHg	0.44	< 0.001	0.38	< 0.001	+ 0.06	+ 14%	0.15
Awake PP, mmHg	0.40	< 0.001	0.36	0.001	+ 0.05	+ 13%	0.25
Asleep PP, mmHg	0.47	< 0.001	0.40	< 0.001	+ 0.07	+ 17%	0.063
AASI	0.37	< 0.001	0.36	0.001	+ 0.01	+ 3%	0.82
SCORE2/SCORE2-OP, %	0.40	0.002	0.37	0.004	+ 0.03	+ 9%	–
Framingham risk score, %	0.41	< 0.001	0.38	0.002	+ 0.04	+ 10%	–

Absolute values of Pearson’s (for ABPM metrics and indices of arterial stiffness) and Spearman’s (for risk scores) correlation coefficients, and absolute and relative differences, with *P* levels of significance

*ABPM* ambulatory blood pressure monitoring, *OSI* overnight stiffness index, *OPPT* overnight pulse propagation time, *SBP* systolic blood pressure, *DBP* diastolic blood pressure, *PP* pulse pressure, *AASI* ambulatory arterial stiffness index

## Discussion

This study on nocturnal PPG in primarily mild-to-moderate hypertensive subjects provides three main findings. First, OSI correlates with well-established cardiovascular risk scores. Second, OSI correlates with blood pressure-derived indices of arterial stiffness. Third, OSI correlates with out-of-office blood pressure obtained by ABPM, in particular during sleep.

Our main finding is that OSI relates to future cardiovascular risk, as assessed by the SCORE2/SCORE2-OP and by the Framingham risk score. This appears to be the first evaluation of overnight PPG-based stiffness index and SCORE2/SCORE2-OP. Our findings are in line with previous studies, where daytime SI [22, 23] and OPPT [26, 27] were associated with cardiovascular risk as assessed by SCORE [31], a previously used cardiovascular risk evaluation score before the introduction of the contemporary SCORE2/SCORE2-OP [5, 6].

In our study, OSI obtained by PPG was correlated with blood pressure-derived indices of arterial stiffness. It is plausible that an increased arterial stiffness, as a measure if vascular ageing, is one explanation behind the association between OSI and cardiovascular risk [10]. The strongest correlation (*r* = 0.47) was observed between OSI and pulse pressure during sleep (i.e. data for both were collected during the same time of the day). These findings for OSI extend results reported for relations between daytime SI and office pulse pressure (r = 0.52) [21]. However, others found only weak, although significant, relations between daytime SI and pulse pressure [23, 24]. We observed a correlation between OSI and AASI, an established marker of cardiovascular risk related to arterial stiffness [12]. This correlation was, however, not retained after adjustment for age and other common risk factors. Previous work has demonstrated a stronger association between pulse pressure and aortic PWV than between AASI and aortic PWV [12, 32]. Of note, studies have shown daytime SI to correlate with aortic PWV (*r* = 0.58–0.66) in healthy subjects, and in cohorts including patients with coronary artery disease, and end stage renal disease [19, 21].

Previous studies have shown correlations between daytime SI and systolic blood pressure [19, 21, 23], and between OPPT and daytime systolic blood pressure [28]. Our results confirm and extend these findings by showing that overnight PPG-based stiffness measurements by OSI are correlated with concurrent ABPM levels, with stronger relations for systolic than for diastolic blood pressure, both during sleep and awake periods. Thus, like established methods for arterial stiffness assessment, OSI is correlated with blood pressure levels [33], and highlights the potential of using measures of propagation and transit times of pulse waves for cuffless estimation of blood pressure [34, 35].

As compared to OPPT, OSI showed stronger correlations with systolic ambulatory blood pressure, systolic blood pressure dipping, and asleep diastolic blood pressure. Further correlations for blood pressure indices were all numerically higher for OSI than for OPPT but these differences did not achieve statistical significance, potentially due to the small sample size. The fact that OPPT was correlated with participants’ height, implies that OSI should be a more useful and correct estimate of arterial stiffness than OPPT.

Several further findings of this study support the notion that OSI may be used to estimate cardiovascular risk. First, OSI was related to a reduced blood pressure dipping pattern which is related to increased cardiovascular risk [36, 37]. Second, OSI was related to increasing age, which extends previous findings with daytime SI [19, 21, 23] and OPPT [28]. Third, OSA is known to be associated with increased cardiovascular risk [38], and in our study OSI was higher in the OSA group, as compared to the group with no OSA, although the relation between OSI and AHI was not retained after adjusting for common confounders. Taken together, these findings suggest that OSI could be used to predict cardiovascular risk. Modern wearable devices providing recordings with higher resolution, in combination with advanced machine learning, may further improve the potential to use OSI and PPG for estimation of cardiovascular risk and vascular ageing [15, 16, 39].

There are some important limitations of this study. First, we acknowledge that assessments of indices of vascular ageing and arterial stiffness by pulse pressure and AASI are subject to several confounding factors. However, both pulse pressure and AASI provide independent information on future risk for cardiovascular events and mortality [11, 12]. Second, cardiovascular risk was assessed by risk scores, but actual outcomes were not assessed. Third, subject-reported times were used to define the sleep period, but we do not have information on the exact sleep period, and to what extent the PPG signal was actually acquired during sleep, or in which sleep stage. Finally, the relatively small study size with more men than women may affect how the results can be generalized.

In conclusion, we show that OSI is an improved PPG feature associated with cardiovascular risk, blood pressure levels, and indices of vascular ageing related to arterial stiffness. Due to the rapidly increasing consumer use of wearable devices with incorporated PPG sensors, further studies to evaluate OSI for cardiovascular risk assessment and management are warranted.

## Supplementary Information

Below is the link to the electronic supplementary material.

Supplementary file1 (DOCX 59 KB)

## Data Availability

The data underlying this article will be shared on a reasonable request and when compatible with current ethical decision and informed consent.
