# Supplementary material for: Overnight stiffness index from finger photoplethysmography in relation to markers of cardiovascular risk and vascular ageing
Source: Heart Vessels. 2025 Mar 14;40(10):895–904. doi: 10.1007/s00380-025-02537-3 (PMC12464147; doi:10.1007/s00380-025-02537-3)
Supplement: Supplementary file 1 — Supplementary file1 (DOCX 59 KB) [file 380_2025_2537_MOESM1_ESM.docx]

**Supplementary information**

Overnight stiffness index from finger photoplethysmography in relation to markers of cardiovascular risk and vascular ageing
Heart and Vessels

Henrik Hellqvist*, Hermine Rietz, Ludger Grote, Jan Hedner, Dirk Sommermeyer, Thomas Kahan, Jonas Spaak
*Corresponding author: Division of Cardiovascular Medicine, Department of Clinical Sciences, Danderyd Hospital, Karolinska Institutet, Stockholm, Sweden; E-mail: henrik.hellqvist@ki.se

Table of contents

[Information about cardiovascular risk scores 2](#_Toc191035203)

[Table S1: Multiple regression and indices of arterial stiffness 3](#_Toc191035204)

[Table S2: Multiple regression and ABPM levels 4](#_Toc191035205)

[Table S3: Characteristics of the study population according to OSA 5](#_Toc191035206)

[Table S4: Multiple regression and AHI 6](#_Toc191035207)

# **Information about cardiovascular risk scores**

**SCORE2 and SCORE2-OP**

SCORE2 is a risk prediction model, which was developed to be used in Europe to estimate the 10-year risk of fatal and non-fatal cardiovascular disease (CVD) in apparently healthy individuals, aged 40–69 years [1]. It is an update of the original SCORE model [2], which only estimated fatal CVD risk. The model for SCORE2 is based on the clinical characteristics age, sex, systolic blood pressure, non-HDL cholesterol and smoking status, and provides the absolute 10-year risk of first onset fatal and non-fatal CVD for four different European risk regions. SCORE2-OP (older people) provides risk estimates for ages 70-89 years, based on the same characteristics as SCORE2 [3].
**Framingham risk score**

The Framingham risk score is a cardiovascular risk prediction model, which was developed from the Framingham Heart Study and is based on a predominantly white US East coast population. The risk model used in the current study [4] estimates an individual’s 10-year risk of developing cardiovascular disease (myocardial infarction, stroke, or cardiovascular death), for ages 30-79 years. The model is based on the clinical characteristics age, sex, systolic blood pressure, total cholesterol, HDL-cholesterol, smoking status, diabetes, and hypertension treatment status.

**References**1. SCORE2 working group and ESC Cardiovascular risk collaboration (2021) SCORE2 risk prediction algorithms: new models to estimate 10-year risk of cardiovascular disease in Europe. Eur Heart J 42(25):2439-2454

2. Conroy RM, Pyorala K, Fitzgerald AP, Sans S, Menotti A, De Backer G, De Bacquer D, Ducimetiere P, Jousilahti P, Keil U, Njolstad I, Oganov RG, Thomsen T, Tunstall-Pedoe H, Tverdal A, Wedel H, Whincup P, Wilhelmsen L, Graham IM, group Sp (2003) Estimation of ten-year risk of fatal cardiovascular disease in Europe: the SCORE project. Eur Heart J 24(11):987-1003

3. SCORE2-OP working group and ESC Cardiovascular risk collaboration (2021) SCORE2-OP risk prediction algorithms: estimating incident cardiovascular event risk in older persons in four geographical risk regions. Eur Heart J 42(25):2455-2467

4. D'Agostino RB, Sr., Vasan RS, Pencina MJ, Wolf PA, Cobain M, Massaro JM, Kannel WB (2008) General cardiovascular risk profile for use in primary care: the Framingham Heart Study. Circulation 117(6):743-753

# **Table S1: Multiple regression and indices of arterial stiffness**

|  | Office PP | | | | 24-h PP | | | | Awake PP | | | | Asleep PP | | | | AASI | | | |
| --- | --- | --- | --- | --- | --- | --- | --- | --- | --- | --- | --- | --- | --- | --- | --- | --- | --- | --- | --- | --- |
|  | B | SE | β | *P* | B | SE | β | *P* | B | SE | β | *P* | B | SE | β | *P* | B | SE | β | *P* |
| (Intercept) | 20.9 | 18 | – | 0.2 | 13.9 | 12 | – | 0.2 | 17.3 | 13 | – | 0.2 | 6.36 | 12 | – | 0.6 | 0.141 | 0.13 | – | 0.3 |
| OSI (m/s) | 1.53 | 0.92 | 0.199 | 0.10 | 1.68 | 0.61 | 0.311 | 0.007 | 1.56 | 0.64 | 0.279 | 0.018 | 1.96 | 0.60 | 0.353 | 0.002 | 0.012 | 0.01 | 0.204 | 0.074 |
| Age (years) | 0.472 | 0.19 | 0.306 | 0.018 | 0.286 | 0.13 | 0.264 | 0.028 | 0.294 | 0.14 | 0.262 | 0.034 | 0.280 | 0.13 | 0.251 | 0.031 | 0.004 | 0.00 | 0.369 | 0.003 |
| Sex (male) | -0.139 | 4.1 | -0.004 | 0.9 | -1.77 | 2.7 | -0.069 | 0.5 | -1.76 | 2.8 | -0.067 | 0.5 | -2.02 | 2.7 | -0.077 | 0.5 | -0.027 | 0.03 | -0.096 | 0.4 |
| BMI (kg/m^2^) | -0.249 | 0.46 | -0.060 | 0.6 | 0.174 | 0.30 | 0.060 | 0.6 | 0.145 | 0.32 | 0.048 | 0.7 | 0.234 | 0.30 | 0.079 | 0.4 | -0.002 | 0.00 | -0.073 | 0.5 |
| Diabetes mellitus (yes) | 2.12 | 7.0 | 0.037 | 0.8 | 6.23 | 4.6 | 0.153 | 0.2 | 5.80 | 4.9 | 0.137 | 0.2 | 7.18 | 4.6 | 0.171 | 0.12 | 0.023 | 0.05 | 0.051 | 0.7 |
| Smoking history (yes) | -2.93 | 3.7 | -0.085 | 0.4 | -1.25 | 2.5 | -0.051 | 0.6 | -1.15 | 2.6 | -0.046 | 0.7 | -1.28 | 2.4 | -0.051 | 0.6 | -0.060 | 0.03 | -0.229 | 0.027 |
| *R*^2^ | 0.14, *P* = 0.011 | | | | 0.24, *P* <0.001 | | | | 0.20, *P* = 0.001 | | | | 0.29, *P* <0.001 | | | | 0.24, *P* <0.001 | | | |

Coefficients unstandardized (B, for 1 unit) with standard error (SE), and standardized (β) for various indices of arterial stiffness, with significance values (*P*) from 78 subjects (1 subject missing smoking history). *P* values for OSI after adjustment for multiple testing: Office PP, 0.10; 24-h PP, 0.028; Awake PP, 0.055; Asleep PP, 0.011; AASI, 0.1. PP, pulse pressure; AASI, ambulatory arterial stiffness index; OSI, overnight stiffness index; BMI, body mass index; *R*^2^, coefficient of determination for the model (adjusted).

# **Table S2: Multiple regression and ABPM levels**

|  | 24-h SBP | | | | Awake SBP | | | | Asleep SBP | | | | 24-h DBP | | | | Awake DBP | | | | Asleep DBP | | | |
| --- | --- | --- | --- | --- | --- | --- | --- | --- | --- | --- | --- | --- | --- | --- | --- | --- | --- | --- | --- | --- | --- | --- | --- | --- |
|  | B | SE | β | *P* | B | SE | β | *P* | B | SE | β | *P* | B | SE | β | *P* | B | SE | β | *P* | B | SE | β | *P* |
| (Intercept) | 87.6 | 15 | – | <0.001 | 98.3 | 16 | – | <0.001 | 60.6 | 16 | – | <0.001 | 73.7 | 8.6 | – | <0.001 | 81.0 | 9.3 | – | <0.001 | 54.2 | 9.0 | – | <0.001 |
| OSI (m/s) | 3.18 | 0.77 | 0.473 | <0.001 | 2.87 | 0.83 | 0.416 | <0.001 | 3.78 | 0.81 | 0.501 | <0.001 | 1.49 | 0.44 | 0.390 | 0.001 | 1.31 | 0.47 | 0.328 | 0.007 | 1.82 | 0.46 | 0.444 | <0.001 |
| Age (years) | -0.025 | 0.16 | -0.018 | 0.9 | -0.029 | 0.17 | -0.021 | 0.9 | 0.050 | 0.17 | 0.033 | 0.8 | -0.311 | 0.09 | -0.406 | 0.001 | -0.324 | 0.10 | -0.403 | 0.002 | -0.229 | 0.10 | -0.279 | 0.021 |
| Sex (male) | 2.38 | 3.4 | 0.075 | 0.5 | 1.87 | 3.7 | 0.057 | 0.6 | 3.88 | 3.6 | 0.109 | 0.3 | 4.16 | 2.0 | 0.230 | 0.036 | 3.62 | 2.1 | 0.191 | 0.088 | 5.90 | 2.0 | 0.304 | 0.005 |
| BMI (kg/m^2^) | 0.392 | 0.39 | 0.109 | 0.3 | 0.374 | 0.42 | 0.101 | 0.4 | 0.455 | 0.41 | 0.112 | 0.3 | 0.217 | 0.22 | 0.106 | 0.3 | 0.228 | 0.24 | 0.106 | 0.3 | 0.223 | 0.23 | 0.102 | 0.3 |
| Diabetes mellitus (yes) | 4.48 | 5.9 | 0.088 | 0.4 | 2.99 | 6.3 | 0.058 | 0.6 | 7.42 | 6.2 | 0.130 | 0.2 | -1.76 | 3.4 | -0.061 | 0.6 | -2.80 | 3.6 | -0.093 | 0.4 | 0.245 | 3.5 | 0.008 | 0.9 |
| Smoking history (yes) | 0.198 | 3.1 | 0.007 | 0.9 | 1.03 | 3.3 | 0.033 | 0.8 | -0.980 | 3.3 | -0.029 | 0.8 | 1.43 | 1.8 | 0.083 | 0.4 | 2.20 | 1.9 | 0.122 | 0.3 | 0.303 | 1.9 | 0.016 | 0.9 |
| *R*^2^ | 0.21, *P* <0.001 | | | | 0.13, *P* = 0.013 | | | | 0.31, *P* <0.001 | | | | 0.19, *P* = 0.001 | | | | 0.16, *P* = 0.005 | | | | 0.24, *P* <0.001 | | | |

Coefficients unstandardized (B, for 1 unit) with standard error (SE), and standardized (β) for various ABPM levels, with significance values (*P*) from 78 subjects (1 subject missing smoking history). *P* values for OSI after adjustment for multiple testing: 24-h SBP, <0.001; Awake SBP, 0.006; Asleep SBP, <0.001; 24-h DBP, 0.007; Awake DBP, 0.029; Asleep DBP, 0.002. ABPM, ambulatory blood pressure monitoring; SBP, systolic blood pressure; DBP, diastolic blood pressure; OSI, overnight stiffness index; BMI, body mass index; *R*^2^, coefficient of determination for the model (adjusted).

# **Table S3: Characteristics of the study population according to OSA**

|  | All | No OSA | OSA | *P* | *Q* |
| --- | --- | --- | --- | --- | --- |
| n | 71 | 20 | 51 | – | – |
| Age, years | 58.6 ± 10.5 | 50.4 ± 10.0 | 61.8 ± 8.9 | <0.001 | 0.003 |
| Sex, male | 50 (70%) | 10 (50%) | 40 (78%) | 0.038 | >0.99 |
| Height, cm | 175 ± 9 | 172 ± 9 | 176 ± 9 | 0.12 | >0.99 |
| Body mass index, kg/m^2^ | 28.1 ± 4.2 | 28.2 ± 5.2 | 28.1 ± 3.8 | 0.97 | >0.99 |
| Smoking history | 26 (37%) | 7 (37%) | 19 (37%) | >0.99 | >0.99 |
| Diabetes mellitus | 7 (10%) | 0 (0%) | 7 (14%) | 0.18 | >0.99 |
| Antihypertensive medication | 36 (51%) | 6 (30%) | 30 (59%) | 0.055 | >0.99 |
| Statin treatment | 22 (31%) | 6 (30%) | 16 (31%) | >0.99 | >0.99 |
| Total cholesterol, mmol/l | 5.34 ± 1.09 | 5.33 ± 0.86 | 5.34 ± 1.16 | 0.98 | >0.99 |
| LDL cholesterol, mmol/l | 3.36 ± 1.02 | 3.45 ± 0.83 | 3.33 ± 1.07 | 0.67 | >0.99 |
| HDL cholesterol, mmol/l | 1.33 ± 0.35 | 1.33 ± 0.29 | 1.33 ± 0.37 | >0.99 | >0.99 |
| eGFR, ml/min/1.73m^2^ | 88 ± 17 | 94 ± 17 | 85 ± 17 | 0.049 | >0.99 |
| SCORE2/SCORE2-OP, % | 6.5 [4.5;10.5] | 5.0 [3.0;6.5] | 7.8 [5.0;12.0] | 0.016 | 0.52 |
| Framingham risk score, % | 17.2 [11.7;27.1] | 10.1 [5.0;20.7] | 19.3 [12.6;27.6] | 0.005 | 0.17 |
| Office SBP, mm Hg | 148 ± 19 | 140 ± 15 | 151 ± 20 | 0.014 | 0.45 |
| Office DBP, mm Hg | 90 ± 11 | 86 ± 10 | 91 ± 11 | 0.058 | >0.99 |
| 24-h SBP, mm Hg | 135 ± 14 | 128 ± 10 | 137 ± 15 | 0.005 | 0.17 |
| Awake SBP, mm Hg | 141 ± 15 | 135 ± 10 | 143 ± 16 | 0.011 | 0.36 |
| Asleep SBP, mm Hg | 121 ± 16 | 113 ± 10 | 125 ± 17 | <0.001 | 0.032 |
| 24-h DBP, mm Hg | 81 ± 8 | 79 ± 6 | 82 ± 9 | 0.092 | >0.99 |
| Awake DBP, mm Hg | 86 ± 9 | 84 ± 7 | 87 ± 9 | 0.18 | >0.99 |
| Asleep DBP, mm Hg | 72 ± 9 | 68 ± 6 | 73 ± 9 | 0.006 | 0.20 |
| Systolic dipping, % | 14 ± 7 | 16 ± 6 | 13 ± 7 | 0.065 | >0.99 |
| Diastolic dipping, % | 17 ± 8 | 19 ± 8 | 16 ± 8 | 0.086 | >0.99 |
| Office PP, mm Hg | 58 ± 17 | 54 ± 13 | 59 ± 18 | 0.13 | >0.99 |
| 24-h PP, mm Hg | 53 ± 11 | 49 ± 9 | 55 ± 12 | 0.042 | >0.99 |
| Awake PP, mm Hg | 55 ± 12 | 51 ± 10 | 57 ± 12 | 0.055 | >0.99 |
| Asleep PP, mm Hg | 50 ± 12 | 46 ± 9 | 52 ± 13 | 0.032 | >0.99 |
| AASI | 0.44 ± 0.12 | 0.40 ± 0.11 | 0.45 ± 0.12 | 0.073 | >0.99 |
| OPPT, ms | 163 ± 38 | 181 ± 37 | 157 ± 36 | 0.016 | 0.51 |
| OSI (m/s) | 11.2 ± 2.2 | 9.8 ± 1.9 | 11.7 ± 2.1 | 0.001 | 0.039 |
| AHI, events/h | 8.6 [4.3;19.0] | 3.0 [1.4;3.8] | 13.7 [8.0;22.3] | <0.001 | <0.001 |

Values presented as mean values ± SD, median values [interquartile range], or n (%), with *P* level of significance between-groups and *Q*, showing adjusted *P*-values after correction for multiple comparisons. OSA, obstructive sleep apnea; eGFR, estimated glomerular filtration rate; SBP, systolic blood pressure; DBP, diastolic blood pressure; PP, pulse pressure; AASI, ambulatory arterial stiffness index; OPPT, overnight pulse propagation time; OSI, overnight stiffness index; AHI, apnea–hypopnea index. n = 71 (8 subjects missing AHI). Diabetes mellitus includes both type 1 and 2.

# **Table S4: Multiple regression and AHI**

|  | AHI | | | |
| --- | --- | --- | --- | --- |
|  | B | SE | β | *P* |
| (Intercept) | -20.6 | 9.9 | – | 0.041 |
| OSI (m/s) | 0.634 | 0.49 | 0.152 | 0.2 |
| Age (years) | 0.269 | 0.11 | 0.304 | 0.017 |
| Sex (male) | 4.47 | 2.2 | 0.222 | 0.047 |
| BMI (kg/m^2^) | 0.269 | 0.25 | 0.121 | 0.3 |
| Diabetes mellitus (yes) | 1.51 | 3.7 | 0.049 | 0.7 |
| Smoking history (yes) | -2.52 | 2.0 | -0.132 | 0.2 |
| *R*^2^ | 0.24, *P* <0.001 | | | |

Coefficients unstandardized (B, for 1 unit) with standard error (SE), and standardized (β) for AHI, with significance value (*P*) from 70 subjects (8 subjects missing AHI and 1 subject missing smoking history). AHI, apnea–hypopnea index; OSI, overnight stiffness index; BMI, body mass index; *R*^2^, coefficient of determination for the model (adjusted).
